# Integrated Biomarker Response for Environmental Assessment Using the Gastropod *Phorcus turbinatus* along the Northern and the Northeastern Coasts of Tunisia

**DOI:** 10.3390/life11060529

**Published:** 2021-06-07

**Authors:** Wafa Boulajfene, Montassar Lasram, Sabiha Zouari-Tlig

**Affiliations:** 1 Laboratoire de Recherche de Biodiversité, Parasitologie et Ecologie des Ecosystèmes Aquatiques, Faculté des Sciences de Tunis Département de Biologie, Université de Tunis El Manar, Tunis 1068, Tunisia; s.zouaritlig@gmail.com; 2 Laboratoire d’Endocrinologie et Physiologie des Agressions, Faculté des Sciences de Tunis Département de Biologie, Université de Tunis El Manar, Tunis 1068, Tunisia; lasram_montassar@yahoo.fr

**Keywords:** oxidative stress, rocky shoreline, contamination, bioindicator

## Abstract

This work aims to assess the spatial and temporal variations of four biomarkers activities and to integrate their biological responses in a battery using the gastropod *Phorcus turbinatus*. The monitoring was carried out during the period between April 2014 and January 2015 at six stations along the northern and the northeastern coasts of Tunisia. The Fulton condition factor was estimated and the activities of catalase, acetylcholinesterase and glutathione-S-transferase were evaluated by spectrophotometry. A multi-biomarker battery approach was used to assess ecosystems’ condition and to identify environmental impacts on the organisms. The results suggest that the enzymatic activities of CAT and GST depend especially on seasons. As for AChE activity, it was similar between seasons and stations. The values of the integrated biological response were maximal at Jarzouna in summer and at Sidi Daoued in winter, indicating the presence of severe stressors suffered by the organisms. This perturbation may be due to the enrichment of the waters by xenobiotics, namely polycyclic aromatic hydrocarbons, insecticides, phosphate wastes, PCBs and pesticides. Thus, *P. turbinatus* seems to be a good bioindicator of chemical pollution, constituting an adequate tool for a bio-monitoring program.

## 1. Introduction

The rocky shoreline shelters a significant richness in species of ecological, patrimonial and economic interest [1]. However, the population explosion and the concentrations of coastal anthropogenic activities, particularly urban, industrial and agricultural activities, expose it to a great quantity pollution of various natures [2]. These environments represent important sources of xenobiotics, including persistent organic pollutants (POPs) and, more specifically, polycyclic aromatic hydrocarbons (PAHs) [3]. These hydrophobic and liposoluble compounds are resistant to photochemical and biological degradation, which promotes their bioaccumulation in the adipose tissues of marine organisms [4]. In addition to POPs, metals, organotin and organometallic compounds and carbon monoxides (CO) damage water and sediment quality [3]. Pyrolytic origin has been identified as the main source of petroleum compounds (PAHs) in coastal sediments of the Mediterranean, and of Tunisia in particular [5,6]. They are derived from the ballasting and dismantling of tankers, oily bilge discharges, refinery effluents, municipal waste, rejected lubricating oils and chronic or accidental oil inputs [6,7,8]. 

The rocky coasts of Tunisia are known to be strongly affected by organostannic compounds (tributyltin TBT) and heavy metals [9,10]. In addition, high total hydrocarbon concentrations were reported in the surface sediment of many areas of the Tunisian coasts, namely Bizerte Jarzouna (602–1270 μg/g) [11], Ghar el Melh lagoon (39.59–655.28 ng/g of dry weight) [12], Bizerta lagoon (83.3–447.08 ng/g of dry weight) [13], the Gulf of Tunis (14.7–618.1 ng/g) [14], Monastir Bay (25.6–576.8 ng/g of dry weight) [8] and the region of Khniss (between 2280 and 7700 μg/g) [15]. Similarly for the Gulf of Gabès, in the eastern Mediterranean zone, [16] showed a high contamination of the surface sediment of the Sfax-Kerkennah canal by polycyclic aromatic hydrocarbons, especially near the port of Sfax (175–10,769 ng/g dry weight of sediment). Note that this contamination was not limited to the sediment but also affected benthic organisms inhabiting those shores. In fact, chemical analysis of the tissue of the mussel *Mytilus galloprovincialis* Lamarck, 1819 [6] showed that tissues from the Gulf of Tunis and the Galite station were strongly affected by total PAHs (68.6 and 69.7 μg/kg). These authors suggested that the waters of the Gulf of Tunis are contaminated with benzo [a] anthracene (8–9.2 μg/kg), chrysene (7.3–9.7 μg/kg), fluoranthene (10.1–11 μg/kg), phenanthrene (8.8–10.3 μg/kg) and pyrene (9.5 μg/kg) [6]. 

According to [17], the exposure of marine organisms to pollutants generates oxidative stress resulting from an imbalance between the production of reactive oxygen species (ROS) and the mechanisms of antioxidant defenses. Thus, alteration of the cellular systems occurs, namely an enzymatic inactivation and a lipid per-oxidation [18]. This is manifested either by binding of the free radicals formed to the soluble or membranous biomolecules of the organism or by reaction of these biomolecules with the thiol groups (SH) [19]. The toxic effects of these radicals are neutralized by the antioxidant enzymes superoxide dismutase (SOD), catalase (CAT), glutathione peroxidase (GSH-Px), glutathione-S-transferase (GST) and glutathione reductase (GR) [20]. In addition, many molecules, such as glutathione (GSH), A, C, and E vitamins can participate in the removal process of oxidizing radicals [17]. Hence, the activities of antioxidant enzymes were widely used as biomarkers for early warnings of aquatic pollution using bioindicator species able to accumulate pollutants [20,21,22]. More specifically, CAT is known forits function of removing hydrogen peroxide during basal metabolism in aerobic organisms but also to be induced by various organic pollutants such as polycyclic aromatic hydrocarbons and polychlorinated biphenyls [19,23]. The activity of GST is often used as a biomarker of organochlorine compounds [24,25]. AChE is involved in synaptic neurotransmission and is often used as a biomarker of exposure to neurotoxic compounds such as organophosphorus pesticides and carbamates [26,27].

The gastropod *P. turbinatus* (Born, 1778) is often considered as a good bioindicator since it is directly linked to the bottom, widespread along the rocky shores and sedentary (low movement) [10,28,29]. However, the potential biological effects of these contaminants remain unknown and little studied. Indeed, no work has focused on the environmental quality monitoring based on biochemical criteria integrating different biomarkers using this species in Tunisia. In order to identify the effect of chemical pollutants, particularly petroleum, on the benthic ecosystem along the coastal northern and north-eastern fringe of Tunisia, it was essential to establish a biomarker battery based on the metabolic response of CAT, GST, AChE and Fulton condition factor K.

The main objectives of this work are (i) to assess the general condition of *P. turbinatus* organisms collected from the northern and the north-eastern coasts of Tunisia by estimating the Fulton condition factor (K) during four seasons, (ii) to study spatiotemporal fluctuations of the metabolic activity in the digestive glands by analyzing four biomarkers (CAT, AChE, GST and Fulton condition factor K), and (iii) to adapt an integrated biological response battery representing the degree of oxidative stress derived from contamination by organic pollutants.

## 2. Material and Methods

**Figure 1:**
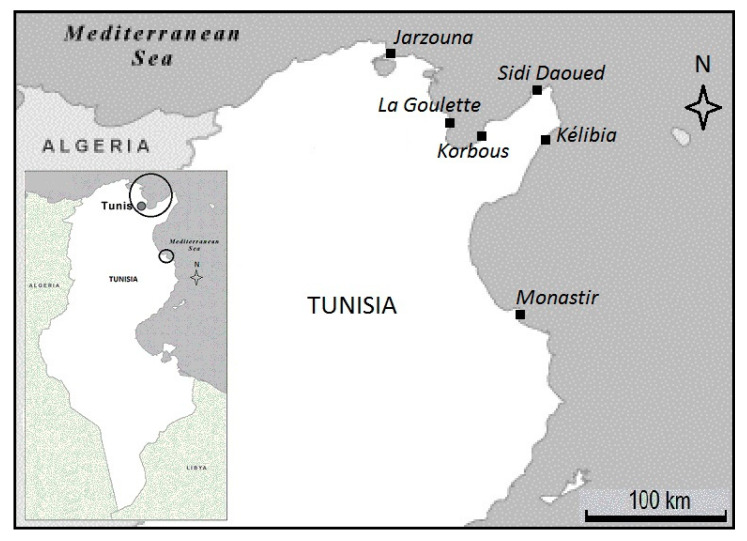
The location of the sampling stations.

In order to estimate the variation of Fulton condition factor and of the biomarker activities in *P. turbinatus*, seasonal monitoring was carried out during the period from April 2014 to January 2015 at six stations (Jarzouna, La Goulette, Korbous, Sidi Daoued, Kelibia and Monastir), along the northern and the northeastern coasts of Tunisia (Figure 1). Note that the choice of this species is justified by its high abundance, its rapid and continuous renewal and its reproductive strategies [30,31]. The stations of Jarzouna (37°15′52.16″ N 9°53′36.05″ E), La Goulette (36°49′08.37″ N 10°18′38.49″ E), Sidi Daoued (37°02′40.03″ N 10°91′09.69″ E), Kelibia (36°83′31.71″ N 11°11′66.47″ E) and Monastir (35°77′34.61″ N 10°83′75.78″ E) are known for their contamination and their proximity to potential sources of pollution (harbors, industrial zones, urban areas) [10]. Korbous station (36°50′31.52″ N 10°34′09.90″ E) was chosen as a reference as it hosts rare and endangered species such as *Patella ferruginea* Gmelin, 1791 and *Pinna nobilis* Linnaeus, 1758 [10]. Moreover, this station showed the minimal sedimentological content in Cu and Cd, the lowest intrinsic Cu, Zn and Hg concentrations in the whole organism of *P. turbinatus* and the lowest mean concentrations of metallothioneins for all seasons except for autumn in the digestive glands of the species [10]. The salinity, the water temperature, the dissolved oxygen level and the potential of hydrogen were recorded in the field by the correspondent portal devices [10].

At each station and season, a sample (n = 10) of adult specimens (size > 1.2 cm) was taken. The organisms were transported to the laboratory alive in sea water. The size (cm) and the total weight (g) were measured for all the sampled individuals which were dissected 6 to 7 h later. The digestive glands, region of pollutants storage, were extracted, weighed and frozen at −80 °C until biochemical analysis. The Fulton condition factor (K) was estimated in the same individuals according to the formula K = Total weight/size^3^ [32]. 

Note that the winter coincides with December, January, and February, the spring with March, April, and May, the summer coincides with June, July, and August, and the autumn coincideswith September, October, and November.

### 2.1. Samples Grinding

A 0.1 g sample of digestive gland was added to 1 mL of PBS (0.1 M, pH = 7.8) and then grounded using an Ultra-Turrax. A centrifugation at 10,000× *g* (10 min at 4 °C) enabled us to obtain a supernatant aliquoted into Eppendorf tubes and stored at −80 °C for consequent biochemical assays of CAT, AChE and GST [33].

### 2.2. Determination of Total Protein

The total protein concentrations within the digestive gland were determined using the Biuret colorimetric method [33,34]. A volume of 20 μL of the glandular sample was added to 1 mL of Gornall reagent (31.9 mmol/L of sodium potassium tartrate, 0.6 mol/L of copper sulphate, 30 mmol/L of potassium iodide, 0.6 mol/L of sodium hydroxide). The whole was incubated at room temperature (20–25 °C), then the optical density was measured at a wavelength λ = 546 nm. The optical density of the standard solution of bovine albumin (50 g/L) was estimated under the same conditions in order to evaluate the total protein concentration in g/L [34].

### 2.3. Catalase

Twenty microlitersof glandular homogenate was mixed with 1 mL of phosphate buffer (0.1 M, 50 mM TRIS, pH = 7.4). The reaction was initiated by the addition of H_2_O_2_. Its evolution was noted every 15 s for one minute by spectrophotometry at a wavelength λ = 240 nm against a white tube containing 1 mL of phosphate buffer [35].

### 2.4. Glutathione-S-Transferase

The GST assay was performed using the method of Habig et al. (1974). Fifty microlitersof sample was mixed with 400 μL of phosphate buffer (0.1 M, 50 mM TRIS, pH 7.4), 530 μL of distilled water and 10 μL of GSH (100 mM). The reaction was initiated by the addition of 10 μL of CDNB (100 mM) to the reaction solution. The optical density is followed per minute for 1 to 4 min at a wavelength λ = 340 nm against a white tube containing the same reagents and considered under the same conditions [36].

### 2.5. Acethylcholinesterase

The determination of the activity of AChE was performed using the method of [37]. The DTNB and the ATCi solutions (0.2 and 3 mM, respectively) were freshly prepared on the analyses day. A volume of 1.8 mL of phosphate buffer, 50 μL of the sample and 50 μL of DTNB were pre-incubated at 37 °C. The addition of 50 μL of ATCi sets off the reaction whose evolution were followed during 4 min at a wavelength λ = 405 nm [37]. For each sample, a control without ATCi substrate showed the possible presence of thiol groups able to react with the DTNB. An enzyme-free control measured the spontaneous hydrolysis of ATCi.

The distribution of the studied biomarkers among seasons and among stations was obtained by the realization of a multiple factorial analysis (MFA) using the XLSTAT software. The correlations between these biomarkers and the physicochemical factors according to seasons and to stations were evaluated by principal component analysis (PCA) using the R_2.14.1_ software. In addition, CAT, GST, AChE and Fulton condition factor k were used to calculate IBR values at each station during the four study seasons and to establish corresponding stars plot.

### 2.6. Integrated Biological Response IBR

Although biomarkers provide valuable information, their use could be limited by a lack of integrated statistical analyses [17,38]. Therefore, it would be necessary to incorporate them into a general framework facilitating data analysis and interpretation in order to provide an integrated relative measure of the overall health of coastal areas. The integrated biological response (IBR) has been applied in numerous field studies in mussels [39], daphnids [38], green crab *Carcinus maenas* [40] and in fish [41,42,43].

The modified multi-biomarker battery approach of [44] was used to assess the health status of ecosystems and to identify the impacts of environmental stress on organisms. The variance of the data was reduced and standardized by the logarithmic transformation $Y_{i}=\log\left(X_{i}/X_{0}\right)$, where X_i_ is the estimated value for a biomarker in an individual i sampled at a given site and X_0_ is the average for a biomarker calculated based on the data used as a reference. The mathematical rules controlling the reduced centered normal law are then applied to the log-transformed data. Thus, the position of each sample compared to the general mean of the considered population is traced by determining the parameter *Z_i_* for each specimen according to the relation $Z_{i}=\frac{Y_{i}-\mu \;}{\sigma },\;$where *μ* is the general mean of a biomarker at a given site and *σ* is the standard deviation. In order to create the baseline adjusted to 0 and to represent the variation of the biomarkers along this line, the normalized average of the biological responses (*Z_i_*) and the mean of the reference data (*Z*_0_) were used to define a gap index of biomarkers (*A_i_*): $A_{i}=Z_{i}-Z_{0}.\;$Thereafter, the absolute values of *A* parameters calculated for each biomarker at each study site are summed as follows: IBR=∑|A|.

For one site, *A* parameters are reported in a star-plot indicating each biomarker deviation compared to the reference line 0. The area exceeding the value 0 reflects the induction of the biomarker and the area below 0 indicates its inhibition. 

## 3. Results

### 3.1. Fulton Condition Factor (K)

**Figure 2:**
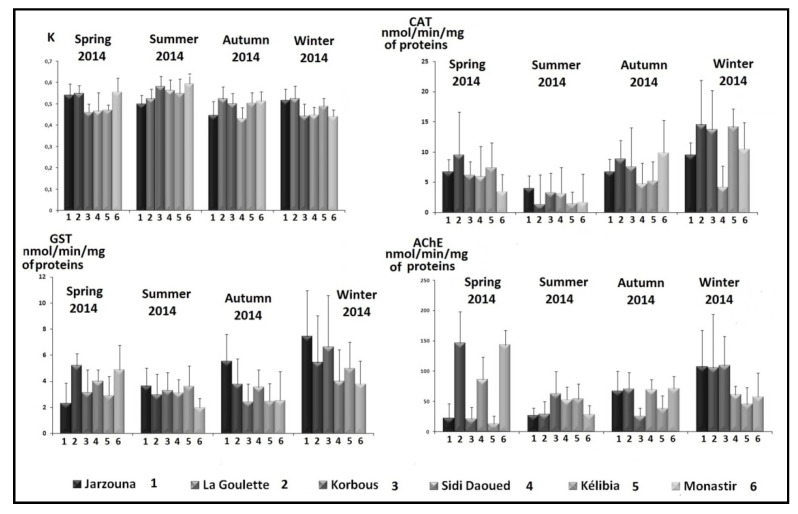
Seasonal evolution of the activity of the four studied biomarkers (spring 2014–winter 2015) in the digestive gland of *P. turbinatus* at the different study stations.

This factor ranges between a minimum of 0.431 estimated in autumn at Sidi Daoued station and a maximum of 0.594 obtained in summer in Monastir (Figure 2). 

### 3.2. Catalase 

The values of the enzymatic activity of CAT oscillate between a minimum recorded at La Goulette during the summer (1.417 ± 4.8 nmol/min/mg of proteins) and a maximum estimated at the same station in winter (14.511 ± 7.35 nmol/min/mg of proteins) (Figure 2). Throughout the whole study period, the evolution of CAT activity showed a decrease in summer followed by a rise in cold seasons. For all the study stations, the minimum values for this activity were recorded in summer, whereas the maximum values were recorded in winter except for Sidi Daoued station, where CAT activity was maximal in autumn. 

### 3.3. Glutathione S-Transferase (GST)

GST activity measured in the digestive gland of *P. turbinatus* oscillates between a minimum of 1.99 ± 0.69 nmol/min/mg of proteins noted at Monastir in summer and a maximum of 7.46 ± 3.49 nmol/min/mg of proteinsestimated at Zarzouna station in winter (Figure 2). The enzyme activity was close during the spring, summer and autumn and reached its maximal values in winter for all the stations except Monastir, which showed a maximum of 4.87 ± 1.88 nmol/min/mg of proteins in the spring. 

### 3.4. Acetylcholinesterase (AChE)

The AChE activity measured in the digestive gland of *P. turbinatus* oscillates between a minimum of 12.76 ± 11.03 nmol/min/mg of proteins noted at Kélibia station in spring and a maximum of 146.28 ± 51.64 nmol/min/mg of proteins recorded at La Goulette station during the same season (Figure 2). 

**Figure 3:**
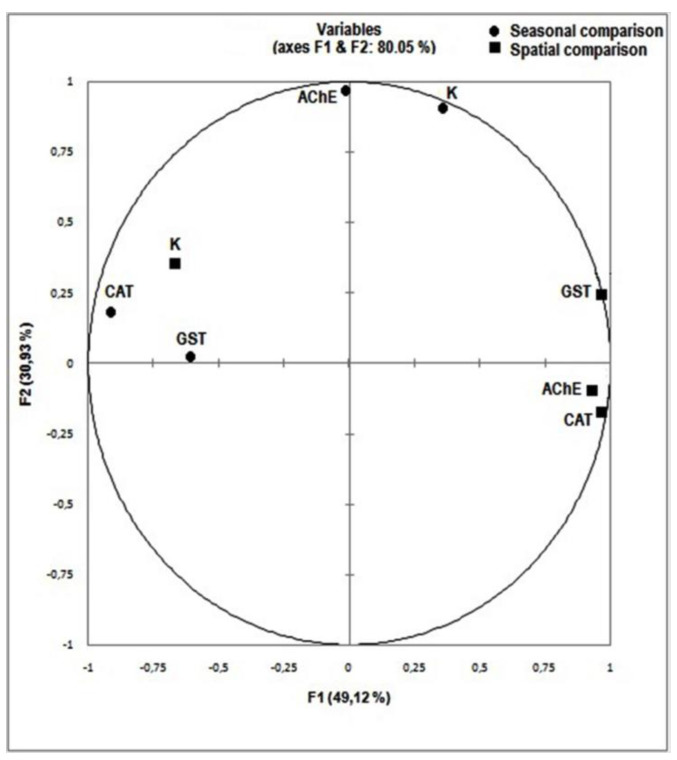
Variables distribution on the first plane (1,2) of MFA depending on spatial and seasonal Variation.

**Figure 4:**
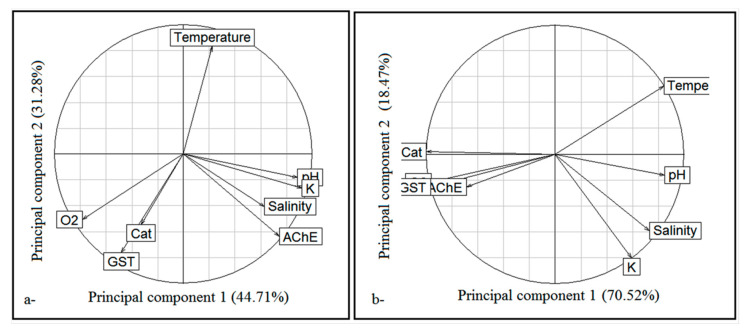
Factorial analysis of the correspondence (FCA) between the biomarkers according to stations (**a**) and to seasons (**b**).

The application of the multiple factorial analyses (MFA) allowed the study of the distribution of the estimated variables (biomarkers) and to compare them among stations and among seasons (Figure 3). This analysis showed that AChE activity was correlated to the Fulton condition factor and that CAT activity was related to GST activity according to seasonal variation. As for the spatial variation, it was shown that the activities of the three enzymes (CAT, AChE and GST) were interconnected. The principal component analysis (PCA) of the studied biomarkers and the physicochemical factors depending on stations revealed that the two first axes explained 75.99% of the total data variability (Figure 4a). The first principal component was represented by K values (0.918) linked to the pH (0.881), to AChE activity (0.743) and to O_2_ values (−0.783). As for the second principal component, it was essentially influenced by the water temperature (0.833) and the GST activity (−0.763). The same analysis performed depending on seasons showed that the two first axes explained 89% of the total data variability (Figure 4b). The first principal component was represented by the temperature (0.842) related to the pH (0.849), to salinity (0.730), to CAT and GST activities (−0.998 and −0.965, respectively) and to O_2_ values (−0.960). As for the second principal component, it was mainly influenced by Fulton condition factor values (−0.803).

### 3.5. Integrated Biological Response IB

The estimation of the integrated biological responses during the four seasons made it possible to evaluate the health state of *P. turbinatus*. This value varies depending on the degree of stress at each station (Table 1). This IBR showed its maximum at Jarzouna station in the summer (7.1014) and at Sidi Daoued station in the winter (7.1307).

**Figure 5:**
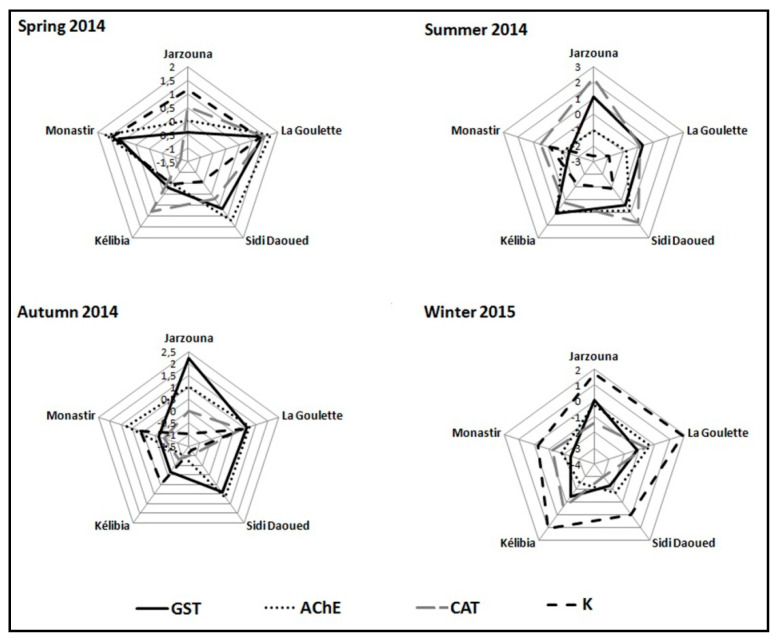
Star-plot of the integrated biological response during the four seasons in *P. turbinatus*.

The star plot (Figure 5) enabled representing the deviation of each studied biomarker compared to the reference station (Korbous).

During the spring, the species showed a low metabolic effort at Kélibia station, while this effort was higher in individuals collected from La Goulette and Monastir explaining a greater stress at these levels. The activity of GST was maximal compared to other biomarkers. 

In summer, the most important biological response occurred at Sidi Daoued and Jarzouna stations, with maximal activity of the CAT enzyme. The metabolic activity of the individuals taken from Kélibia was mainly dominated by GST whereas those from La Goulette showed a low metabolic effort absorbed mainly by CAT.

The snails collected during the autumn were characterized by a strong biological response of the GST, especially at Jarzouna station.This activity was less important for the other biomarkers at the remaining stations. As for Sidi Daoued and La Goulette stations, the biological response of the organisms was mainly explained by GST and AChE activities. 

During the winter, the metabolic effort of *P. turbinatus* species was maximal at La Goulette station, dominated essentially by Fulton condition factor (K). This effort devoted to the increase was lower in the individuals from Sidi Daoued and Monastir. As for the integrated biological response of the individuals from Kelibia, it was mainly absorbed by the Fulton condition factor and the CAT and GST activities (Figure 5).

## 4. Discussion

This work aims to diagnose the current state of the benthic ecosystem along the northern and the northeastern coast of Tunisia under pressure from chemical pollutants, and more specifically petroleum. To do this, a seasonal evaluation of four biomarkers activities was performed in the gastropod *P. turbinatus*. We found that the study species may be a good bioindicator of organic pollution, constituting a suitable material for a marine bio-monitoring program. Many other studies [3,38,39] reported the usefulness of antioxidants as early warning systems on ecosystems’ health. These non-specific metabolic responses depend on the intensity and the duration of the oxidative stress supported by the organisms. Thus, antioxidant parameters may serve as biomarkers of complex marine contamination using marine invertebrates [45]. 

The Fulton condition factor is one of the most commonly used biomarkers of initial selection. It was chosen as a preliminary biomarker of selection to provide data about energy storage varying according to the life cycle of the organism, the physicochemical and nutritional factors.It also constitutes an indicator of exposure to environmental toxicants [46]. In this study, the reported seasonal variation of this factor suggests that the metabolic effort devoted to the growth of the organisms depends on the environmental factors and the general state of oxidative stress characterizing each season. In fact, the Fulton condition factor (K) was maximal in summer and minimal in winter, indicating that *P. turbinatus* appears to be more sensitive to oxidative stress and to live in unbalanced metabolic states during these seasons. The similarity of this factor depending on stations (Figure 2) could be related to trophic availability in all the study localities [10], in particular the *RhodophyceaeNemalion helminthoides* and *Rissoella verruculosa*, potential foods of this gastropod [47].

According to [19], the activity of CAT, indicating the degree of cells alteration, depends on the physicochemical factors of the environment, especially temperature and dissolved oxygen. This correlation seems to justify the catalytic activity noted in the present work which was minimal in summer and maximal in winter. These results corroborate those of [48] in *P. articulatus* species taken from the same station with a CAT activity ranging between 0.82 ± 1.97 nmol/min/mg of proteinsat Sidi Daoued station in summer and 15.46 ± 9.41 nmol/min/mg of proteinsestimated at La Goulette in autumn. Additionally, [3] suggested stimulation of CAT activity in the goby *Pomatoschistus microps* taken from Portugal during the cold seasons (autumn and winter). This enzyme is also sensitive to various organic contaminants, including pesticides and chemical fertilizers known to increase its activity [49,50]. In fact, the use of pesticides is increased in the north and northeast sector of the country where farms and agricultural lands are concentrated [49]. Moreover, this fringe is jointly contaminated by urban discharges and hydrocarbons evacuated in harbors by transiting ships [9]. In this context, [45,51,52] reported that CAT activity was often induced by exposure to PAHs, COs and PCBs. In addition, a significant increase in CAT activity was observed in the digestive gland of mussels exposed to paraquat (herbicide) [53] and to menadione (naphthoquinone) [54]. Note that long exposure to petroleum compounds may lead to CAT response reduction due to superoxide radical fluxes which prevent catalytic activity [55]. These data could explain the low activity of this enzyme at Sidi Daoued compared to the other stations and the decrease in its activity during the spring and the summer characterized by an intense maritime traffic. The catalytic activities recorded in this study were much lower than those recorded by [56] and by [57] in *Ruditapes decussatus* taken from Bizerta lagoon (80 and 100 nmol/min/mg of proteins, respectively). 

The activity of GST is generally induced by organochlorine compounds (DDT, dieldrin, chlordane, heptachlor, etc.) [24] but it could also be accelerated by metals, particularly copper. Indeed, this biomolecule is provided with an adaptive and protective role against the oxidative stress caused by these substances [58]. It responds to the accumulation of metals by the production of superoxide ions which induce superoxide dismutase (SOD) to transform the superoxide radical into H_2_O_2_ [58]. Therefore, the high enzymatic activities of GST reported in the present study at Jarzouna, La Goulette and Sidi Daoued stations during the autumn seem to be related to high doses of copper [10] and of organochlorine compounds [6,14]. In addition, these areas are subject to phosphate waste discharge from the electrical transformers sites in Menzel Bourgiba, Tunis and Beni Khiar (Nabeul) and polyurethane-chloro biphenyl (PCB) sites in Bizerte, Tunis, Nabeul and Monastir [59]. The low GST responses recorded at Jarzouna and Kélibia stations in the spring and at Kélibia in the autumn may be related to the high concentrations of toxic substances that could inhibit or even deactivate GST activity [45]. As for [39], they reported a negative correlation between GST activity and salinity by means of experimental tests using mussels. This could explain, in our case, the low activity of this biomarker in the study monodont observed in summer in comparison with other seasons at all the stations [10]. The assay of this enzyme, in the present work, showed values (1.99 ± 0.69–7.46 ± 3.49 nmol/min/mg of proteins) similar to those estimated by [48] in *P. articulatus*(between a minimum of 2.35 ± 0.84 nmol/min/mg of proteinsnoted at La Goulette in winter and a maximum of 6.49 ± 4.75 nmol/min/mg of proteinsestimated at Kelibia station in autumn),but lower than those estimated by [29] in the mantle of *P. turbinatus* taken from the eastern coasts of Algeria (1.2 ± 0.14–21.9 ± 14.84 μMol/mn/mg of proteins). Our estimates were also much lower than those of [40,57,60]. These latter recorded a GST activity of about 500 nmol/min/mg of proteins in the digestive gland of *Ruditapes decussatus* (Bizerta Lagoon). [40] also reported strong enzymatic activity of GST in *Carcinus maenas* taken from six sites in Monastir Bay (between 70 and 180 μmol/min/mg protein). As for [60], they assessed very high doses of GST in the digestive glands of *Perna perna* (350 nmol/min/mg of proteins) and *Mytilus galloprovencialis* (360 nmol/min/mg of proteins) collected from the Bay of Agadir (South of Morocco). 

In invertebrates, AChE, a general physiological stress marker [49,61], is inhibited by insecticides, including permethrin (neurotoxin), which causes acetylcholine accumulation in the synaptic space [62]. This action maintains a permanent transmission of the nerve impulses leading generally to the muscular tetany and to the death of the organism [63]. In addition, [39] related the inhibition of AChE in transplanted mussels from a control to a polluted site (Bay of Cannes) to the high contamination of the site by copper. For this purpose, the low enzymatic response of this enzyme observed in the present work in La Goulette, Sidi Daoued and Monastir during the summer could be due to the high Cu concentration at these levels [10] and to the increased use of insecticides in the agricultural lands surrounding these coasts [62,64]. The activity of this enzyme was similar at all the study stations, with a minimum noted in Korbous. These recorded values are considerably lower than those estimated by [29] (11.75 ± 1.7 μMol/min/mg of proteins to 80 ± 19.57 μMol/min/mg of proteins) and higher than those reported by Kamel et al. (2012) in the digestive gland of *Ruditapes decussatus* taken from an aquaculture farm at Menzel Jemil (Tunisia) (ChE: 1.1–1.5 nmol/min/mg of proteins).

It should be noted that integrated biological response (IBR) analysis make it possible to understand the contribution of the various biomarkers to the metabolic response of this trochus and to assess the degree of stress depending on stations and on seasons. It was proved that the stations of Jarzouna (summer 2014) and of Sidi Daoued (Winter 2014) seem to be the sites that are most affected by pollutants. This appears to reflect the presence of high stress suffered by these organisms. Indeed, these two zones are subject to several industrial and urban stressors and include large fishing ports with continuous maritime activity [9]. The maximal IBR values were synchronized with the minimal values of the Fulton condition factor (K), probably related to the reproductive activity of organisms during the summer and the autumn [30]. These results corroborate those of [3], who reported high biological activity when Fulton condition factor values were low, probably in relation to the reproductive status of *Pomatoschistus microps* taken from Portugal. In our case, the biological response was minimal during spring and autumn, mainly due to the activity of GST. This seems to indicate a physiological equilibrium in the studied organisms during these two seasons, which are characterized by a strong algal presence and a decrease in the concentrations of toxic substances [10]. 

However, anthropogenic inputs and/or environmental conditions are not the only phenomena involved in the metabolic response. Populations collected from different stations may exhibit a large genetic polymorphism and not respond in the same way to the ecosystem changes. Indeed, the enzymatic activity could also vary according to their sexual maturity and their physiological state [19]. According to [65], it seems that the selection of an appropriate battery of biomarkers could avoid incorrect answers obtained with a single biomarker and would summarize the information in a set of multivariate data [65]. Note that the value of the IBR, which is a mathematical relationship, becomes increasingly credible with the increase in the number of used biomarkers and the decrease in their relative weights [66]. For this reason, it would be essential to raise the number of biomarkers in future bio-surveillance programs for a better understanding of the physiological state of the organisms in a given ecosystem.

## 5. Conclusions

This work constitutes a preliminary investigation of the activities of four biomarkers in *P. turbinatus* along the rocky coasts of the northern and the northeastern parts of Tunisia. According to the obtained results, the study species seems to be a good bioindicator of organic pollution, constituting a suitable material for a marine bio-monitoring program. Moreover, the studied biomarkers responses do not depend on stations and seem to fluctuate according to seasons except for AChE. This appears to reflect the intensities of contamination and possible interactions between pollutants varying according to physicochemical factors. Indeed, the stress reported in these monodonts seems to be derived from the enrichment of the waters of the northern and the northeastern coasts of Tunisia with toxic pollutants, namely polycyclic aromatic hydrocarbons, insecticides, phosphate wastes, PCBs and pesticides. Overall, the IBR Index provided an integrated view of the biological effects of pollution and classified the health status of the organisms in coastal areas while discriminating seasonal patterns of contamination. In addition, our selected biomarkers seem to correspond with the objectives of the ecotoxicological study by being either early warning signals (GST, CAT, AChE) or adverse effects of chemical exposure (Fulton condition factor K). However, this work deserves to be extended in space, towards the south of the country (Gulf of Gabes), and in time, with a monthly or even daily monitoring of the environmental factors to confirm or affirm the absence of spatial variation reported in the present work. Moreover, the number of combined biomarkers needs to be increased in order to obtain a better representation that is more useful for the decision-makers.

## Figures and Tables

**Table 1:** IBR values in *P. turbinatus* during the four seasons at the different study stations.

Stations	Spring 2014	Summer 2014	Autumn 2014	Winter 2015
Jarzouna	2.1235	**7.1014**	4.2104	3.4296
La Goulette	5.8597	3.1587	4.0036	3.9436
Sidi Daoued	2.6256	4.0106	4.4448	**7.1307**
Kélibia	2.0367	3.5005	2.5134	5.6174
Monastir	5.5657	4.0106	2.4611	5.7923
